# Co(II) Coordination in Prokaryotic Zinc Finger Domains as Revealed by UV-Vis Spectroscopy

**DOI:** 10.1155/2017/1527247

**Published:** 2017-12-14

**Authors:** Valeria Sivo, Gianluca D'Abrosca, Luigi Russo, Rosa Iacovino, Paolo Vincenzo Pedone, Roberto Fattorusso, Carla Isernia, Gaetano Malgieri

**Affiliations:** Department of Environmental, Biological and Pharmaceutical Science and Technology, University of Campania-Luigi Vanvitelli, Via Vivaldi 43, 81100 Caserta, Italy

## Abstract

Co(II) electronic configuration allows its use as a spectroscopic probe in UV-Vis experiments to characterize the metal coordination sphere that is an essential component of the functional structure of zinc-binding proteins and to evaluate the metal ion affinities of these proteins. Here, exploiting the capability of the prokaryotic zinc finger to use different combinations of residues to properly coordinate the structural metal ion, we provide the UV-Vis characterization of Co(II) addition to Ros87 and its mutant Ros87_C27D which bears an unusual CysAspHis_2_ coordination sphere. Zinc finger sites containing only one cysteine have been infrequently characterized. We show for the CysAspHis_2_ coordination an intense *d*-*d* transition band, blue-shifted with respect to the Cys_2_His_2_ sphere. These data complemented by NMR and CD data demonstrate that the tetrahedral geometry of the metal site is retained also in the case of a single-cysteine coordination sphere.

## 1. Introduction

Metal ions in protein complexes exert many fundamental biological functions spanning from a simple structural role to direct participation in catalytic activities [1, 2]. Metalloproteins are, in fact, very abundant, and many of the biological metals have *d*-orbital electrons that consent them to experience different oxidation states. Moreover, transition metals allow *d*-orbital hybridization in complex with ligands and thus coordination of more ligands and a variety of coordination geometries [3]. In the different protein sites, metal ions can be found bound to endogenous (both backbone and side chain atoms of the polypeptide) or exogenous ligands (i.e., other molecules bound to the protein) [4, 5]. Many protein-bound metals are divalent ions, and the affinity evaluation of the protein for the metal has been the object of numerous studies [6–11]. The affinities measured in different buffers and at different pH values evidence their dependence upon the measurement conditions as well as the method used for the analysis. Affinity for a given metal ion, both native and exogenous, is certainly an essential information for metalloproteins' complete characterization, and whatever be the used technique, it is well known that it crucially depends on the set of coordinating amino acids.

Among the metalloproteins and metal-binding domains, the zinc finger motif, characterized by the presence of a structural zinc ion, is surely the most emblematic [12–15] as it has been intensively studied for its known ubiquitous presence in the biological world (e.g., 3% of the genes of the human genome encode for zinc fingers containing proteins [16, 17]).

The zinc finger family is made up of several members that bind zinc with a different combination of cysteines and histidines. In the classical eukaryotic zinc fingers, also named “Kruppel ZF,” two cysteines and two histidines bind zinc with high affinity. Four cysteine coordination sites and sites constituted by three Cys and one His can also be found to tightly bind the structural zinc ion, with this coordination being always essential for the domain folding [12–15].

The DNA-binding domain of the prokaryotic Cys_2_His_2_ zinc finger protein Ros (Ros87) folds in a domain that is structurally different and significantly larger than its eukaryotic counterpart. Ros87, held together by the structural zinc and by a 15-residue hydrophobic core, consists of 58 residues arranged in a βββαα topology [18]. Numerous Ros homologues (Ros/MucR family) have been identified [14,19–24], in which the coordination sphere appears to be composed of only one (the first) cysteine [19]. The second coordinating residue is usually an aspartate, indicating for this domain the possibility of a CysAspHis_2_ coordination. The structural characterization of Ros87_C27D [25], an Ros87 mutant with an aspartate in the second coordinating position, has demonstrated that this residue surrogates the role of the second cysteine by monodentally coordinating the zinc ion; this mutation only slightly perturbs the functional structure of the domain.

The main issue when characterizing a zinc protein/peptide interaction is that Zn(II) is a *d*10 ion, spectroscopically silent. So, if a structural change accompanies the binding, folding or unfolding [8] can be followed with circular dichroism (CD) or nuclear magnetic resonance (NMR), but in general, the most diffuse procedure to evaluate the zinc ion affinities considers a fully Co(II)-loaded protein and follows the Co(II) displacement by zinc via UV-Vis spectroscopy [6,15,26–30].

Cobalt(II), being a *d*7 ion used as a probe, can substitute the native metal into both structural and catalytic metal-binding sites of the examined proteins. Co(II) and Zn(II) are nearly the same size [31] (ionic radius of 0.58 Å and 0.60 Å, resp.), and many zinc-binding sites have been shown to be metal substitutable [32–35]. In some cases, enzymes with a catalytic zinc site have been shown to have similar or even higher enzymatic activity when Co(II) substitutes native Zn(II) [3].

Upon Co(II) coordination of ligands, a splitting of the energy levels of *d*-orbital electrons occurs. The Co(II)-ligand system absorbs light at specific wavelengths owing to the so-called *d*-*d* transitions, that is, the excitation and relaxation of the *d*-orbital electrons [36]. The nature and number of coordinating ligands together with the overall coordination geometry of the system dictate the wavelengths and the intensities at which this absorption occurs [37, 38]: an intense band (*ε* > 300 M^−1^ cm^−1^) at 625 ± 50 nm is diagnostic of a tetrahedral coordination and a weak band (*ε* ≤ 30 M^−1^ cm^−1^) at 525 ± 50 nm reveals an octahedral complex. An intermediate band (50 ≤ *ε* ≤ 250 M^−1^ cm^−1^) indicates a penta-coordination [26].

Co(II) gives absorption bands also at different wavelengths: due to the S^−^ → Co(II) ligand-to-metal charge transfer (LMCT), an intense absorption band in the near UV, between 316 and 340 nm, can be observed. This band is very useful as the magnitude of the extinction coefficient at 320 nm permits to infer the number of S^−^-Co(II) bonds as each bond contributes to *ε* by about 900–1200 M^−1^ cm^−1^ [39, 40]. Summarizing, while the *ε* at ∼320 nm can count the number of S^−^ involved in the coordination, the *ε* at ∼600 nm is utilized to detect the coordination geometry and to hypothesize the nature of the other ligands [26].

Here, exploiting the capability of the prokaryotic zinc finger to use different combinations of residues to properly coordinate the structural metal ion [18, 25, 41], we describe the effect of Co(II) binding on the larger prokaryotic zinc finger domain Ros87 and on one of its mutant Ros87_C27D.

## 2. Materials and Methods

### 2.1. Protein Expression and Purification

All the proteins used were expressed and purified as previously reported [42]. Only freshly prepared samples were used in all experiments. Briefly, the _pet_Ros56-142 (Ros87) and _pet_Ros56-142_C82D (Ros87_C27D) proteins were produced as follows: ^15^N labeling for NMR experiments was achieved by growing the cells at 37°C in a modified minimal medium containing ^15^NH_4_Cl as the sole nitrogen source, while for UV-Vis and circular dichroism experiments, the proteins were expressed in LB medium. In both cases, the protein expression was induced for ∼2.0 h with 1.0 mM IPTG.

The cells were then harvested, suspended in 20 mM Na_2_HPO_4_ (pH 6.8) buffer, and lysed by sonication. The crude cell extracts were purified by centrifugation, and the supernatant was applied to a Mono S HR 5/5 cation exchange chromatography column (Amersham Biosciences). The pooled fractions containing the proteins were applied to a HiLoad 26/60 Superdex 75 (Amersham Biosciences) gel filtration chromatography column.

### 2.2. UV-Vis Spectroscopy

The native zinc ion was removed obtaining apoRos87 and apoRos87_C27D by acidifying to pH 2.5 the protein solutions in the presence of 150 µM TCEP using HCl 0.1 M and dialyzing against 10 mM Tris, 150 µM TCEP, pH 2.5. The pH was finally readjusted to 6.5, and it has been strictly controlled throughout the experiments. UV-Vis spectra for the Co(II) addition experiments to Ros87 and to apoRos87_C27D were recorded in 10 mM Tris, 20 µM TCEP, pH 6.5, on a Shimadzu UV-1800 spectrophotometer in the range of 200–800 nm at room temperature. The apoprotein solution (4 μM in the case of Ros87 and 3 μM in the case of Ros87_C27D) has been titrated with aliquots corresponding each to an increase of 0.4 μM of final Co(II) concentration in solution for each step. 0.1 mM CoCl_2_ solution was used up to 1.6 Co(II)/protein ratio. Each experiment has been repeated at least three times obtaining comparable results. Protein concentrations were obtained using absorption at 280 nm at pH 2.5.

### 2.3. NMR Spectroscopy

NMR samples contained 150 μM of proteins in 10 mM Tris and 150 µM TCEP at pH 6.5 in the presence of 1.4 equivalents of CoCl_2_ and 90% H_2_O/10% ^2^H_2_O. All the HSQC spectra were recorded at 298 K on a Bruker Avance III HD 600 MHz equipped with cryoprobe at the Department of Environmental, Biological and Pharmaceutical Science and Technology, University of Campania-Luigi Vanvitelli (Caserta, Italy). ^1^H and ^15^N chemical shifts were calibrated indirectly by using TMS as external references. All NMR spectroscopy data were processed with the TopSpin 3.5 software (Bruker) and analyzed by using the computer-aided resonance assignment [43] (CARA) software (downloaded from cara.nmr.ch).

### 2.4. Circular Dichroism

Circular dichroism experiments were collected using a JASCO J-815 CD spectropolarimeter equipped with Peltier temperature control. Data were collected in the 200–260 nm wavelength range using a quartz cuvette with a 1 cm pathlength, with a data pitch of 1 nm, a band width of 1 nm, and a scanning speed of 50 nm/min. All CD samples contained ∼15 μM of proteins in 10 mM Tris and 150 µM TCEP at pH 6.5. A fresh solution of CoCl_2_ 5.0 mM has been used to reach a final [Co^2+^]/[protein] ratio of 1.4. All the spectra were acquired in duplicates and were subtracted from the buffer contribution. Spectra deconvolution has been performed using the server BeStSel [44].

## 3. Results and Discussion

**Figure 1:**
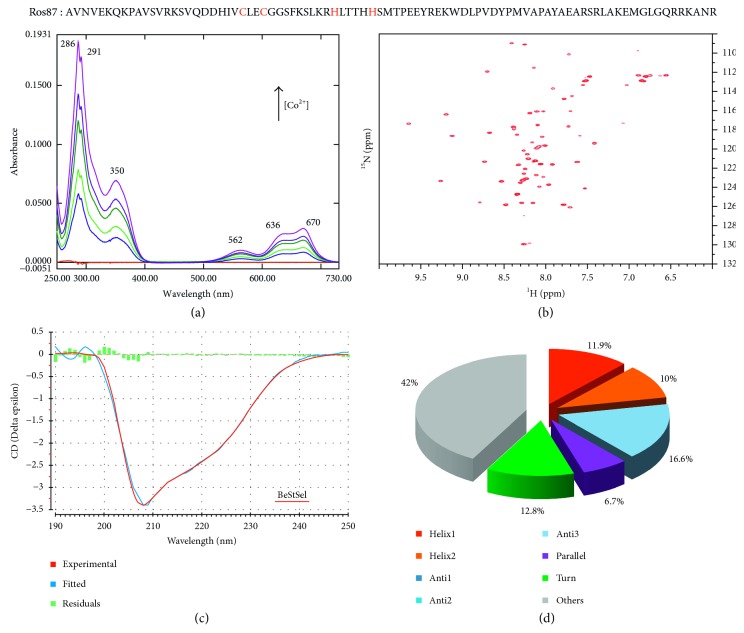
(Top) Ros87 amino acid sequence; (a) UV-Vis spectra of Ros87 titration with CoCl_2_; (b) the ^1^H-^15^N HSQC spectrum of Ros87 in the presence of 1.4 equivalents of Co(II); (c) the experimental CD spectrum of Ros87 (red) overlaid to the fitted CD data (blue) by the server BeStSel; the green histogram indicates the deviations; (d) secondary structure content calculated from the CD data by the server BeStSel.

**Figure 2:**
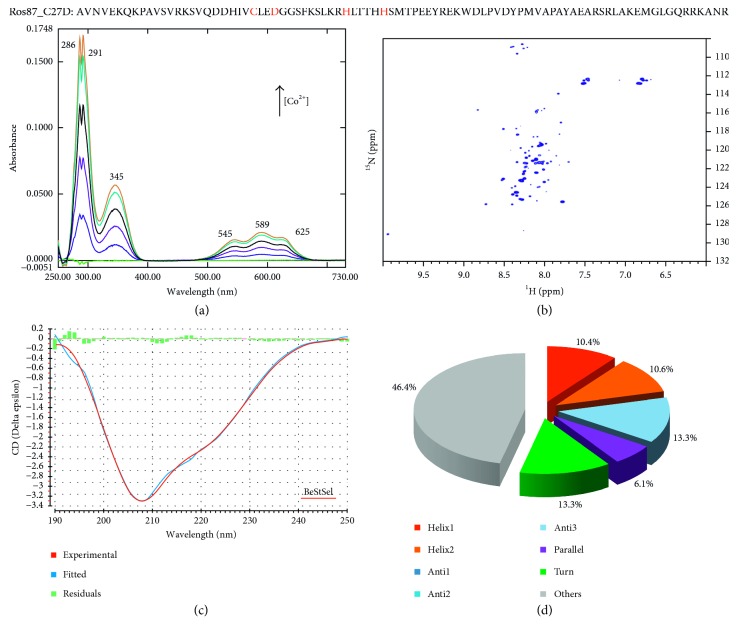
(Top) Ros87_C27D amino acid sequence; (a) UV-Vis spectra of Ros87 titration with CoCl_2_; (b) the ^1^H-^15^N HSQC spectrum of Ros87 in the presence of 1.4 equivalents of Co(II); (c) the experimental CD spectrum of Ros87_C27D (red) overlaid to the fitted CD data (blue) by the server BeStSel; the green histogram indicates the deviations; (d) secondary structure content calculated from the CD data by the server BeStSel.

The UV-Vis spectra of the titration of apo-Ros87 (i.e., the unfolded prokaryotic zinc finger Ros87 with no native Zn(II) bound) and apo-Ros87_C27D (i.e., Ros87 with the second coordinating cysteine mutated in aspartate) with CoCl_2_ are shown in Figures 1 and 2.

In the case of Co(II)-Ros87, the *ε* value in the near UV (at ∼320 nm) that reflects the number of thiolate groups coordinated is 1950 M^−1^ cm^−1^ at 350 nm, indicating that the protein uses two thiol groups to coordinate with Co(II) ion. On the other hand, the *ε* value for Co(II)-Ros87_C27D is 1020 M^−1^ cm^−1^ at 345 nm, indicating the involvement of one thiol group in Co(II) coordination. In both cases, the lack of changes in the shape of the spectrum and in the wavelength of the transition during the titration permits to exclude the formation of complexes with different protein/Co(II) ratios (i.e., 2 : 1, 3 : 1, or more) formed at low Co(II) concentrations [28]. This UV-Vis behaviour was previously independently seen on the same proteins in HEPES buffer [25].

Intense absorption bands around 589–670 nm are also observed for both proteins. These results indicate that Ros87 coordinates the Co(II) with a tetrahedral geometry. Also, Co(II)-Ros87_C27D exhibits an intense *d*-*d* absorption band centered at about 589 nm with the *ε* value of 380 M^−1^ cm^−1^ indicating also in this case a tetrahedral geometry.

Accordingly, Figures 1 and 2 show the two ^1^H-^15^N HSQC spectra of Ros87 and Ros87_C27D, respectively, in the presence of 1.4 equivalents of Co(II) ion. Both spectra show a combination of intense and discrete signals in both proton and nitrogen dimensions indicating the interaction of Ros87 and Ros87_C27D with the paramagnetic Co(II), which gives rise in both cases to folded conformations with stable tertiary structures (Co(II)-Ros87 and Co(II)-Ros87_C27D). Importantly, the two spectra show a meaningful overlap with the holo-Ros87 spectra (data not shown) in the regions not influenced by the paramagnetism of Co(II), thus suggesting for the cobalt-loaded proteins a structure very similar to the zinc-loaded proteins.

Accordingly, the CD spectra indicate that also the secondary structure content of both proteins appears to be well conserved in the Co(II)-loaded structures with respect to the zinc-loaded conformations (Figures 1 and 2). In fact, both CD spectra are characteristic of well-structured proteins containing both α-helical and β-sheet secondary structure. We estimated from the CD data the protein secondary structure for the two proteins using the server BeStSel (Figures 1 and 2). This server fits the CD experimental curve by linearly combining fixed basis components to get the percentage of the eight secondary structural elements [44]. The data indicate that Co(II)-Ros87 and Co(II)-Ros87_C27D structures have a content of secondary structure similar to that of the Ros87-calculated structure (PDB code 2JSP) and Ros87_C27D computational model [25] as determined using the software MOLMOL [45] and DSSP [46, 47].

The data reported here overall indicate for the Co(II) complexation a tetrahedral coordination geometry similar to that of the native zinc and that the replacement of the zinc ion by the Co(II) does not drastically perturb the structural properties of the prokaryotic zinc finger domain.

**Figure 3:**
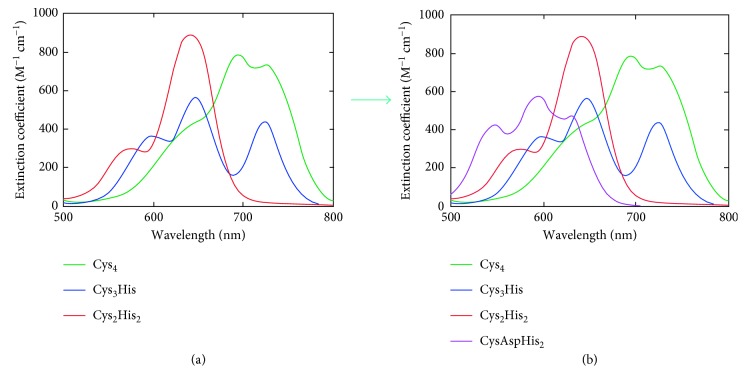
(a) Scheme of the UV-Vis spectra in the 500–800 nm range for Co(II) tetrahedral coordination by different ZFs: four Cys (green line), three Cys and one His (blue line), and two Cys and two His (red line) [37, 49]. (b) Introducing the single-cysteine coordination sphere in the scheme (violet line). The shape of the transition pattern is extremely sensitive to the structure [50].

Interestingly, the comparison of the UV-Vis spectra of Ros87_C27D with those reported in literature for zinc fingers with Cys_2_His_2_, Cys_3_His, and Cys_4_ coordination outlines a blue shift of the *d*-*d* transition bands of the protein that uses a single cysteine to coordinate the metal ion [48] (Figure 2). This shift is in agreement with what has been reported by Krizek et al. [10], who describe increasing shifts of the *d*-*d* transition to higher energies as the number of coordinating cysteines decreases. UV-Vis spectra of zinc finger metal sites containing only one cysteine have been rarely reported [48]. In the eukaryotic Cys_2_His_2_ ZF, the substitution of the second cysteine may result in some cases (i.e., the substitution with an aspartate or with a glutamate [48]) in coordination geometries different than the native tetrahedral coordination demonstrated by weak *d*-*d* absorption bands. Here, we found an intense band at 589 nm that, together with NMR and CD data, indicates a tetrahedral coordination of the metal ion with a resulting blue shift of the *d*-*d* absorption bands. We therefore propose that the scheme of the spectra of tetrahedral coordination of Co(II) in zinc fingers with different numbers of cysteines and histidines (Figure 3) [37, 49] can be implemented (Figure 3) with our results.

**Figure 4:**
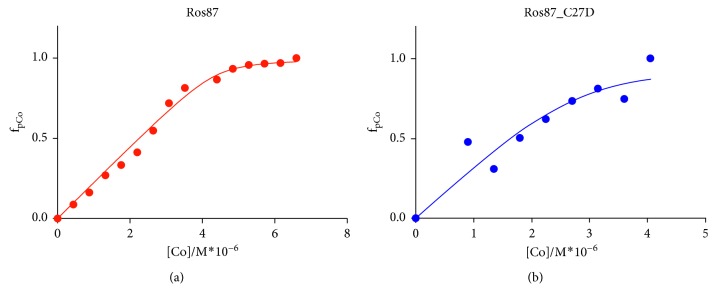
(a) Titration of Ros87 with CoCl_2_ monitored by recording the absorbance at 352 nm. The absorbance is plotted against cobalt concentration. (b) Titration of Ros87_C27D with CoCl_2_ monitored by recording the absorbance at 346 nm. The absorbance is plotted against cobalt concentration.

We also determined the affinities of two proteins for Co(II), through direct titrations in Tris buffer at pH 6.5 which shows that complexes definitively form when the Co(II)/protein molar ratio was equal to 1.4. Using the 1 : 1 model to fit the UV data (Figure 4) [30], we obtain a lower limit for the β constant of 5.59 (±1.97) × 10^−8^ for Ros87 and 2.35 (±0.92) ×10^−7^ for Ros87_C27D.

The successive titration of the Co(II)-loaded proteins with Zn(II) induces a progressive reduction of both bands; the disappearance upon addition of a twofold excess of Zn(II) ion compared with Co(II) indicates that a Co(II) ion was substituted with the spectroscopically inert Zn(II) ion.

## 4. Conclusions

In this article, we report the spectroscopic and structural characterization of the Co(II)-substituted forms of the prokaryotic zinc finger Ros87, a native zinc protein, and of its mutant Ros87_C27D in which the second coordinating cysteine is mutated to aspartate. UV-Vis spectra of zinc finger sites containing only one cysteine, neither regarding zinc, nor other metals of interest, have been rarely reported [48]. In the case of the eukaryotic Cys_2_His_2_ zinc finger, the substitution of the second coordinating cysteine may result in some cases (i.e., the substitution with an aspartate or with a glutamic acid [48]) in a coordination geometry different than the native tetrahedral coordination demonstrated by weak *d*-*d* absorption bands; when a histidine substitutes the cysteine, the coordination remains tetrahedral. Here, we show that, in the prokaryotic domain, the substitution of the native zinc with cobalt mutation does not profoundly affect the structure of the domain and that the substitution of the second ligand amino acid with an aspartate gives rise to an intense band at 589 nm that indicates how this substitution does not markedly change the tetrahedral coordination geometry of the metal ion. We also show how the presence of a single cysteine in the coordination sphere of the protein implies strong *d*-*d* absorption bands in the UV-Vis spectra, blue-shifted with respect to the two cysteines coordination.

Differently from what happens for the small eukaryotic domain, our data outline how in the case of larger proteins like Ros87_C27D, other elements composing the structure (e.g., large hydrophobic cores) play a determinant role in determining the geometry of the coordination sphere. Overall, the UV-Vis spectroscopy confirms to be an excellent and extremely sensitive tool to determine the number and geometry of ligands in structural metal sites.
